# Comparison between clinical characteristics and laboratory findings among patients with complicated and noncomplicated SARS‐CoV‐2 infection: A single‐center experience from Shebin Al‐Kom, Egypt

**DOI:** 10.1002/iid3.671

**Published:** 2022-07-12

**Authors:** Walaa A. Fadda, Manal A. Al‐Batanony, Reham E. E. Aboukhalil, Heba F. Khader, Osamah Al Rugaie

**Affiliations:** ^1^ Department of Basic Medical Sciences, Unaizah College of Medicine and Medical Sciences Qassim University Unaizah Kingdom of Saudi Arabia; ^2^ Department of Human Anatomy and Embryology, Menoufia Faculty of Medicine Menoufia University Shebin Al‐Kom Egypt; ^3^ Department of Family and Community Medicine, Unaizah College of Medicine and Medical Sciences Qassim University Unaizah Kingdom of Saudi Arabia; ^4^ Department of Public Health and Community Medicine, Menoufia Faculty of Medicine Menoufia University Shebin Al‐Kom Egypt; ^5^ Department of Medical Biochemistry, Menoufia Faculty of Medicine Menoufia University Shebin Al‐Kom Egypt

**Keywords:** COVID‐19, laboratory indicators, mortality, severity

## Abstract

**Background:**

Coronavirus disease 2019 (COVID‐19) infection is considered a serious highly infectious disease caused by severe acute respiratory syndrome coronavirus 2, resulting in more than 6.27 million deaths worldwide.

**Aim of the study:**

The study aimed to compare clinical characteristics and laboratory findings of COVID‐19 patients with complications and without complications and discriminate the important risk factors for the complications and deaths.

**Subjects and Methods:**

This cross‐sectional study included 75 confirmed COVID‐19 positive patients; out of which 49 were severely‐ill cases. Analysis of all patients' clinical and laboratory information on admission including serum ferritin, thrombotic activity (d‐dimer), lactate dehydrogenase (LDH), C‐reactive protein (CRP), creatinine, aspartate aminotransferase, and alanine aminotransferase were done.

**Results:**

Lymphopenia, tachycardia, tachypnea, elevated CRP, d‐dimer, serum ferritin, LDH, and decreased SpO_2_ were significantly associated with complicated cases (*p* < .05 for all). By using multivariate logistic regression analysis models, elevated serum ferritin and tachycardia were significantly correlated with the increased odds of complicated COVID‐19 cases (odds ratio [confidence interval 95%] = 10.42 [2.32–46.89] and 8.01 [1.17–55.99]; respectively) (*p* = .002 and .007, respectively).

**Conclusion:**

Lymphocytopenia, d‐dimer, LDH, and CRP levels, which were significantly linked to the severity of COVID‐19, were the prognostic biomarkers to predict the disease severity.

## Graphical abstract

This study linked the prognostic biomarkers to predict coronavirus disease 2019 (COVID‐19) disease severity, hence our decision to submit it to your journal. We aimed to compare COVID‐19 patients with and without complications regarding their clinical characteristics and laboratory findings and discriminate the important risk factors for the complications and death. This cross‐sectional study was carried out in a monocentric secondary healthcare hospital in Menoufia Governorate, Egypt, during the period between May 1st and June 31st, 2021. A convenient nonprobability sample of 75 patients, who were suspected clinically and confirmed by positive reverse transcription‐polymerase chain reaction as COVID‐19 cases, was recruited. Analysis of their clinical and laboratory information on admission including serum ferritin, thrombotic activity (d‐dimer), lactate dehydrogenase (LDH), C‐reactive protein (CRP), creatinine, aspartate aminotransferase, and alanine aminotransferase were done. Lymphocytopenia, tachycardia, tachypnea, elevated CRP, d‐dimer, serum ferritin, LDH, and decreased SP0_2_ were significantly observed in cases with complicated severe acute respiratory syndrome coronavirus 2 (*p* < .05 for all). By using a multivariate logistic regression analysis model, elevated serum ferritin and tachycardia were significantly correlated with the increased odds of complicated COVID‐19 (odds ratio [confidence interval 95%] = 10.42 [2.32–46.89] and 8.01 [1.17–55.99], respectively) (*p* = .002 and .007; respectively). Lymphocytopenia, d‐dimer, LDH, and CRP levels, which were significantly linked to the severity of COVID‐19, were the prognostic biomarkers to predict the disease severity.

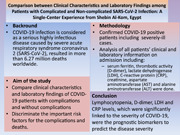

## 1 INTRODUCTION

Worldwide infectious pneumonia of unknown cause has emerged in Wuhan City, China, in December 2019. The causative virus was quickly detected and was labeled as a novel coronavirus (severe acute respiratory syndrome coronavirus, SARS‐CoV‐2). The disease was subsequently called coronavirus disease 2019 (COVID‐19).^1^

Till May 2022, the World Health Organization (WHO) reported a global spread of 521,920,560 confirmed COVID‐19 cases, with 6,274,323 deaths.^2^ The Egyptian confirmed cases of COVID‐19 reported to the WHO, was estimated to be 513,881 where deaths were 24,690 in the period between January and May 2022.^3^

The manifestations of COVID‐19 infection vary among patients in different areas but the symptoms reported in mild‐to‐moderate nonhospitalized cases are mainly headache, loss of smell, nasal obstruction, asthenia, and ear, nose, and throat symptoms.^4^ The principal manifestations reported on hospital admission are mainly fatigue, cough, and fever.^5^, ^6^ However, gastrointestinal disorders are less commonly stated by patients.^5^

Even though most of the patients with COVID‐19 improved after treatment, it was stated that 6.1% of the patients deteriorated into critical conditions, and those patients represented about 85% of all patients who passed away.^7^ The complexity in the pathogenesis of this disease affects different body systems and still not clear in many aspects. Many clinical proofs supported that patients responded to the infection by developing an unusual inflammatory response, resulting in multiple organ failures that ended in death.^8^ Critical cases showed exaggerated dyspnea and hypoxemia that may be followed by resistant metabolic acidosis, septic shock, and acute respiratory distress syndrome, and were rapidly deteriorated to coagulation dysfunction.^9^

In the fight against COVID‐19 disease severity and patients' mortality, the prognostic factors should be detected as early as possible, thus better management strategy could be ensured. In a recent Chinese study about predicting indicators and pathogenesis of critical COVID‐19 cases, they concluded that disease progression warning factors, including biochemical (e.g., aspartate aminotransferase [AST] and alanine aminotransferase [ALT]), hematological (e.g., white blood cell [WBC] count and lymphocyte count), inflammatory (e.g., C‐reactive protein [CRP]), and coagulation (e.g., d‐dimer) biomarkers can increase clinical efficacy, delay the progression of mild/moderate to severe/critical disease, and lowering mortality rates.^10^

Inflammatory markers, including CRP, WBCs, fibrinogen, lactate dehydrogenase (LDH), and d‐dimer, were frequently detected in laboratories to evaluate sepsis development.^11^, ^12^ Lately, data also showed that iron metabolism played an important role in predicting patients' admission to intensive care units (ICUs) and even mortality. To predict COVID‐19 patients' mortality, serum ferritin was reported as a predictor parameter.^13^

The routine investigations included kidney function tests, electrolytes, liver function tests, creatine kinase, LDH, complete blood count (CBC), and coagulation profile.^1^ The ABO system of blood grouping revealed genetic polymorphisms. This gene was related to different traits, and one of them was the increased rate of morbidity and mortality when infected with COVID‐19.^14^

This study aimed to evaluate the prognostic markers for COVID‐19 severity and identify the indictors for complications and mortality among those patients.

## 2 METHODOLOGY

### 2.1 Study design and sampling

This cross‐sectional study was carried out in a monocentric secondary health care hospital in Menoufia Governorate, Egypt, during the period between May 1st and June 31st, 2021. A convenient nonprobability sample of 75 patients, who were suspected clinically and confirmed by positive reverse transcription‐polymerase chain reaction (PCR) as COVID‐19 cases, was recruited. Exclusion criteria included patients suspected clinically but had negative PCR results, cases with incomplete clinical and laboratory data due to either death or early discharge from the hospital, or cases with special conditions including pregnancy, cancer patients, patients under immunosuppressive treatments, patients with chronic liver disease, and patients with acute coronary syndrome. The demographic and clinical data as well as laboratory parameters were collected from the registered medical records.

### 2.2 Data collection

On hospital admission, the following data were collected:

A. Sociodemographic data as, age‐group in years, sex, occupation, smoking status, and source of exposure to COVID‐19 virus (if known).

B. Presence of any comorbidity; hypertension, diabetes mellitus, asthma, or obesity.

C. Clinical manifestations including signs and symptom of COVID‐19 disease.

D. Laboratory investigations include CBC, erythrocyte sedimentation rate, AST, ALT, CRP, urea (blood urea nitrogen), albumin, creatinine, d‐dimer, serum ferritin, LDH, and blood grouping.

### 2.3 Severity assessment

Cases were categorized as mild, ordinary, severe, and critical cases on the basis of “The Guidance for Corona Virus Disease 2019: Prevention, Control, Diagnosis, and Management edited by the National Health Commission of the People's Republic of China.”^15^

(1) “Mild cases had mild clinical symptoms and no pneumonia manifestations on imaging.” (2) “Ordinary cases had symptoms like fever and respiratory tract symptoms, and pneumonia manifestations which can be seen in imaging.” (3) “Severe cases that met any of the following findings: respiratory distress, respiratory rate (RR) ≥ 30 breaths/min; the oxygen saturation percentage (SpO_2_%) less than 93% in resting‐state; or arterial partial pressure of oxygen PaO_2_/oxygen concentration FiO_2_ ≤ 300 mmHg (1 mmHg = 0:133 kPa).” (4) “Critical cases that met any of the following findings: respiratory failure, and mechanical ventilation is required; shock occurs; or complicated with any organ failure that requires monitoring and treatment in ICU.”

In this study, the patients were categorized into two subgroups:

A) Noncomplicated cases group (26 patients): Patients showing mild and common symptoms are described as (1) and (2).

B) Complicated cases group (49 patients): Severe or critically severe cases including patient's criteria in (3) and (4).

The minimum required sample size was calculated as being 52 patients (26 for each group) with a power of 0.8, effect size as 0.8, α‐error as 0.05, and allocation ratio *n*2/*n*1 as 1. Approval to direct this survey was granted by the Deanship of Scientific Research, Qassim University with Grant No.10037‐L‐1‐1‐2020.

### 2.4 Data management and analysis plan

To tabulate and analyze the data, the IBM program with Statistical Package for the Social Sciences of version 25 was used (SPSS Inc., 2011; IBM SPSS statistics for windows, version 20.0; IBM Corp.). Quantitative data were presented as mean ± standard deviation. Student *t*‐test or Mann–Whitney tests were used for comparing two groups of normally distributed or non‐normally distributed variables, respectively. Qualitative data were displayed as frequency distribution (*n* and %) and the *χ* ^2^ test was applied for comparison. Multivariate logistic regression analysis models were performed to ascertain the influence of possible determinants on the outcome (disease complications or mortality). A significant level was considered at two tailed *p* ≤ .05.

## 3 RESULTS

The mean recorded age of the 75 COVID‐19 cases was 62.5 ± 13.3, ranging from 17 to 86 years. Comorbidities were reported in two‐thirds of the cases, with obesity and hypertension the most cardinal (69.3% and 64%, respectively), followed by diabetes mellitus (54.7%), then asthma (25.3%). Among the studied COVID‐19 patients, 49 patients presented with severe and critical complications (65.3%). Their mean age was 63.2 ± 12.5, out of which 71% were obese, 65% were hypertensive, 59% were diabetic, 33% were asthmatic, and 51% were smokers. About three‐fourths (74%) out of the 49 complicated cases were admitted to the ICU, one‐fifth (20%) were intubated and nearly one‐third (31%) died during hospitalization (Tables 1 and 2).

**Table 1:** Sociodemographic data and comorbidity among studied complicated and noncomplicated cases groups

Sociodemographic data	Complicated cases (*n* = 49)	Noncomplicated cases (*n* = 26)	*p*
Age (mean ± SD)	63.2 ± 12.5	61.1 ± 14.8	.51*
Sex No (%)			.78**
Female	21 (43)	12 (46)	
Male	28 (57)	14 (54)	
Occupation No (%)			.17**
No job/homemaker	10 (20)	8 (31)	
Working	22 (45)	6 (23)	
Retired	17 (35)	12 (46)	
Source of exposure to infection No (%)			
At work	13 (26)	4 (15)	.19**
From a family member	16 (33)	14 (54)	
Not known	20 (41)	8 (31)	
HTN No (%)	32 (65)	16 (62)	.75**
DM No (%)	29 (59)	12 (46)	.28**
Asthma No (%)	16 (33)	3 (12)	**.04** **
Smoking No (%)	25 (51)	8 (31)	.09**
Obesity No (%)	35 (71)	17 (65)	.59**

*Note*: Bold values indicates statistically significant.

Abbreviations: DM, diabetes mellitus; HTN, hypertension.

* *t*‐test.

** *χ* ^2^ test.

**Table 2:** Clinical manifestations among studied groups

Manifestations	Complicated cases (*n* = 49)	Noncomplicated cases (*n* = 26)	*p*
Cough No (%)	42 (86)	22 (85)	.89*
Dyspnea No (%)	41 (84)	16 (62)	**.03** *
Fatigue No (%)	44 (80)	20 (69)	.13*
Myalgia No (%)	36 (74)	16 (62)	.29*
Chest tightness No (%)	34 (69)	11 (42)	**.02** *
Fever No (%)	39 (68)	18 (32)	.32*
Headache No (%)	32 (65)	16 (62)	.75*
Sputum No (%)	27 (55)	16 (62)	.59*
Coma No (%)	22 (45)	0 (0)	**<.001** *
Diarrhea No (%)	20 (41)	11 (42)	.9*
Days from illness onset till dyspnea (mean ± SD)	3.6 ± 2.6	3.2 ± 2.9	.52**
ICU admission No (%)	36 (74)	0 (0)	**<.001** *
Death No (%)	15 (31)	1 (4)	**.007** *
Intubation No (%)	10 (20)	0 (0)	**.01** *

*Note*: Bold values indicates statistically significant.

Abbreviation: ICU, intensive care unit.

* *χ* ^2^ test.

** Mann–Whitney *U* test.

There was nonsignificant difference between complicated and noncomplicated COVID‐19 cases regarding their sociodemographic characteristics (age, sex, source of infection, and occupation; *p* > .05). Although most of the complicated COVID‐19 patients were males (57%), but on comparison with those in the noncomplicated group (54%) there was nonsignificant difference (*p* = .07). Regarding the presence of comorbidities, asthma was significantly more prevalent among complicated COVID‐19 cases (33%) than noncomplicated ones (12%) (*p* = .04) (Table 1). Most of the clinical symptoms were more frequent between severe/critical cases reaching a significant level for chest tightness, coma, and dyspnea (69%, 45%, and 84%, respectively) than mild/ordinary ones (42%, 0%, and 62%, respectively) (Table 2).

SpO_2_% and RR were reported as criteria of advanced COVID‐19 condition, and it was obvious that complicated COVID‐19 cases had significant worse mean value of SpO_2_% (83.6 ± 7.2) and elevated mean value of RR (29.04 ± 6.6) than noncomplicated group (95.8 ± 1.8 and 20.0 ± 4.5, respectively) (*p* < .001 for both). Moreover, the mean values of heart rate (HR), d‐dimer, CPR, LDH, and serum ferritin were significantly higher among severe/critical COVID‐19 cases (93.9 ± 17.1, 1.9 ± 1.7, 49.8 ± 27.9, 812.7 ± 445.8, and 621.6 ± 228.7) than mild/ordinary group (71.5 ± 8.3, 1.1 ± 1.0, 15.7 ± 12.8, 355.6 ± 105, and 239.6 ± 84.9; respectively) (*p* < .001, .007, <.001, <.001, and <.001, respectively). Lymphocytopenia was significantly prevalent among complicated COVID‐19 cases (1.95 ± 2.15) than others (5.22 ± 2.82) (*p* < .001) (Table 3).

**Table 3:** Vital signs, laboratory investigations, and radiological findings among the studied groups

Investigations	Complicated cases	Noncomplicated cases (*n* = 26)	*p*
	(*n* = 49)		
Highest temperature °C	38.4 ± 0.9	37.9 ± 0.9	.054*
HR beats/min	93.9 ± 17.1	71.5 ± 8.3	**<.001** *
RR breaths/min	29.04 ± 6.6	20.0 ± 4.5	**<.001** *
SpO_2_%	83.6 ± 7.2	95.8 ± 1.8	**<.001** *
HB (g/dl)	11.7 ± 1.2	11.4 ± 0.9	.21*
WBCs (× 10^3^/µl)	11.76 ± 3.4	11.43 ± 3.7	.82**
Lymphocytic count (× 10^3^/µl)	1.95 ± 2.15	5.22 ± 2.82	**<.001** **
Neutrophils (× 10^3^/µl)	78.4 ± 8.3	80.2 ± 4.5	.31*
PLTs (10^3^/µl)	221.4 ± 54.9	225.4 ± 48.9	.76*
D‐dimer (mg/L)	1.9 ± 1.7	1.1 ± 1.0	**.007** **
ESR mm/h	32.1 ± 17.6	31.4 ± 16.8	.84**
CRP (mg/L)	49.8 ± 27.9	15.7 ± 12.8	**<.001** **
LDH (IU/L)	812.7 ± 445.8	355.6 ± 105	**<.001** **
Serum ferritin (ng/ml)	621.6 ± 228.7	239.6 ± 84.9	**<.001** **
AST (U/L)	47.1 ± 32.1	41.3 ± 22.1	.44**
ALT (U/L)	47.3 ± 34.0	42.3 ± 21.3	.53**
BUN (mmol/L)	22.1 ± 9.4	21.1 ± 7.9	.91**
Albumin (g/dl)	3.7 ± 0.6	3.7 ± 0.61	.89*
Creatinine (mmol/L)	1.3 ± 0.68	1.2 ± 0.43	.42*
Blood grouping: No (%)			.18***
A	11 (22)	1 (4)	
B	5 (10)	2 (8)	
AB	2 (4)	2 (8)	
O	31 (64)	21 (80)	

*Note*: Bold values indicates statistically significant.

Abbreviations: ALT, alanine aminotransferase; AST, aspartate aminotransferase; BUN, blood urea nitrogen; CRP, C‐reactive protein; ESR, erythrocyte sedimentation rate; HB, hemoglobin; HR, heart rate; LDH, lactate dehydrogenase; PLTs, platelets; RR, respiratory rate; SpO_2_%, oxygen saturation percentage; WBCs, white blood cells.

* *t*‐test.

** Mann–Whitney *U* test.

*** *χ* ^2^ test.

The significant univariate risk factors which were indicators for COVID‐19 severity included elevated d‐dimer, CPR, serum ferritin, lymphocytopenia, and tachypnea (odds ratio, OR [confidence interval, CI 95%] = 5.77 [1.59–20.94], 4.45 [1.93–21.6], 20 [5.94–67.3], 0.06 [0.007–0.46], and 0.04 [0.008–0.18], respectively) (*p* = .008, .002, <.001, .007, and <.001, respectively). The variables which were still significantly correlated with the increased odds of complicated COVID‐19 while applying the multivariable logistic regression model were elevated serum ferritin and tachycardia (OR [CI 95%] = 10.42 [2.32–46.89] and 8.01 [1.17–55.99], respectively) (*p* = .002 and .007, respectively) (Table 4). On performing univariate analysis, elevated serum ferritin, low SpO_2_%, tachycardia, and tachypnea were found to be significantly associated with COVID‐19 mortality (*p* = .04, .03, .02, and .04, respectively). However, none of those variables were significantly associated with the increased odds of COVID‐19 mortality while applying the multivariable logistic regression model (*p* > .05) (Table 5).

**Table 4:** Risk factors associated with complicated cases among COVID‐19 patients

Risk factors	Univariate		Multivariate	
	OR (CI 95%)	*p*	OR (CI 95%)	*p*
Asthma: Yes versus no	0.27 (0.07–1.03)	.055	–	–
d‐dimer				
<1	1 (ref)			
1–2	5.77 (1.59–20.94)	**.008**	2.94 (0.47–18.38)	.25
>2	1.48 (0.34–6.48)	.59	0.92 (0.11–7.76)	.94
CRP				
≤10	1 (ref)			
>10	4.45 (1.93–21.6)	**.002**	1.86 (0.37–9.38)	.45
Serum ferritin				
≤307	1 (ref)			
>307	20 (5.94–67.3)	**<.001**	10.42 (2.32–46.89)	**.002**
LDH				
<280	1 (ref)			
≥280	3.38 (0.86–13.29)	.08	–	**–**
HR				
≤85	1 (ref)			
>85	0.04 (0.008–0.18)	**<.001**	8.01 (1.17–55.99)	**.03**
SpO_2_%				
≥93	1 (ref)			
<93	5.52 (1.15–26.46)	.99	–	–
Lymphocytic count				
≥1	1 (ref)			
<1	0.06 (0.007–0.46)	**.007**	0.53 (0.04–6.34)	.61

*Note*: Bold values indicates statistically significant.

Abbreviations: CI, confidence interval; COVID‐19, coronavirus disease 2019; CRP, C‐reactive protein; HR, heart rate; LDH, lactate dehydrogenase; OR, odds ratio; RR, respiratory rate; SpO_2_%, oxygen saturation percentage.

**Table 5:** The risk factor associated with mortality among COVID‐19 patients

Risk factors	Univariate		Multivariate	
	OR (CI 95%)	*p*	OR (CI 95%)	*p*
d‐dimer				
<1	1 (ref)			
1–2	1.47 (0.41–5.33)	.56		
>2	1.5 (0.36‐6.29)	.58	–	**–**
CRP				
≤10	1 (ref)			
>10	5.11 (0.62–42.01)	.13	–	–
Serum ferritin				
≤307	1 (ref)			
>307	0.19 (0.04–0.93)	**.04**	2.39 (0.31–18.31)	.40
LDH				
<280	1 (ref)			
≥280	0.91 (0.17–4.76)	.91	–	–
HR				
≤85	1 (ref)			
>85	4.4 (1.26–15.19)	**.02**	2.72 (0.46–16.2)	.27
RR				
<30	1 (ref)			
≥30	3.25 (1.03–10.23)	**.04**	0.7 (0.12–4.12)	.69
SpO_2_%				
≥93	1 (ref)			
<93	5.52 (1.15–26.46)	**.03**	2.28 (0.28–18.2)	.44
Lymphocytic count				
≥1	1 (ref)			
<1	0.4 (0.13–1.27)	.12	–	**–**

*Note*: Bold values indicates statistically significant.

Abbreviations: CI, confidence interval; COVID‐19, coronavirus disease 2019; CRP, C‐reactive protein; HR, heart rate; LDH, lactate dehydrogenase; OR, odds ratio; RR, respiratory rate; SpO_2_%, oxygen saturation percentage.

## 4 DISCUSSION

To date, COVID‐19 infection represents a continuing challenge for all medical field specialties to explore and understand it more deeply in all its aspects and invade the mystery to final prevention or treatment.

The current study aimed to focus on the characteristics of complicated COVID‐19 patients and compare them with mild/ordinary cases and evaluate the biomarkers and risk factors for disease severity or even death.

In this study, although male patients showed a higher percentage of complications than females it did not reach a significant level. This is in agreement with Huang et al.^16^ who assumed that the frequency of infected COVID‐19 patients was nearly the same among their male and female patients. Moreover, the mean age of our severely‐ill patients was higher than that for the less‐severely affected ones; however, a significant level was not reached. This finding is in line with Starke et al.^17^ who concluded that after adjustment for major age‐dependent risk factors, the age had only a minor impact on COVID‐19 disease severity and death.

Although there was a low frequency of asthmatic patients among the total studied COVID‐19 positive cases representing 25.3% (19/75), asthma was significantly more prevalent among severe/critical COVID‐19 patients (33%) than mild/ordinary ones (12%). During early stages of COVID‐19 pandemic, Chinese^18^, ^19^ and Italian studies^20^, ^21^ conflictingly reported asthma as a risk factor significantly associated with COVID‐19 infection. However, a systematic review and meta‐analysis done by Terry et al.^22^ including 150 studies showed no evidence of increased risk of COVID‐19 infection among asthmatics. Asthma by itself does not increase the risk for COVID‐19 infection in general, but its association with other factors, such as smoking and presence of other comorbidities, makes these patients more vulnerable to worst outcomes.^19^ In accordance, Williamson et al.^23^ while investigating the database from the United Kingdom reported the association of severity of COVID‐19 infection and asthma observing the records of 17 million patients with COVID‐19 infection. After race and sex adjustment, a higher mortality rate of their hospitalized COVID‐19 patients showed significant association with severe asthma.

Our results showed a significantly lower mean value of lymphocytic count between the severe/critical COVID‐19 patients group when compared to the noncomplicated COVID‐19 patients. This is in alignment with Huang et al.^24^ who registered that most of their COVID‐19 patients had low lymphocyte counts. One study revealed that the prominent distinguishing test of severely ill cases of COVID‐19 infection was lymphopenia, which was the result of lymphocytes destruction mainly CD8‐T and CD4‐T lymphocytes, or destruction of cytokine‐mediated lymphocytes.^25^ Furthermore, lymphopenia <1× 10^3^/μl appeared as a significant risk factor for COVID‐19 disease severity on univariate analysis. This means that hospitalized COVID‐19 cases who were not admitted to the ICU, and have a total lymphocytic count of less than 1000/μl, might be in a real need of serious intervention, even with the absence of critically severe manifestations as being in an increased risk for further deterioration of their condition as was confirmed by Zheng et al.^26^

In the current study, most markers of inflammation in laboratory findings were increased among severely‐ill cases. The COVID‐19 infection basic pathophysiology in severe patients is related to the consequences of the cytokine storm. The presence of cytokine storm in those patients with decreased lymphocyte count may represent the uncontrolled progression of the virus observed in severe cases.^27^ Overactivated immune response leads to cytokine storm which is strongly linked to lymphopenia may be by enhancing apoptosis by proinflammatory cytokines. The angiotensin‐converting‐enzyme 2 receptors expressed by lymphocytes may be a direct target of SARS‐CoV‐2 infection.^28^

In this study, the COVID‐19 complicated patients' group showed a significantly higher CRP, LDH, and serum ferritin than noncomplicated COVID‐19 group. This is in agreement with other studies conducted in China^29^, ^30^ where a significantly elevated LDH level among COVID‐19 patients who needed ICU admission was found in comparison with those who did not need ICU care. In Egypt, Doghish et al.^31^ explained that with increasing COVID‐19 infection severity, cytokine‐mediated lung damage occurs with the release of more LDH as well as patients with severe lung damage release more LDH in the circulation. Our study analysis revealed a significant elevation of d‐dimer among the complicated COVID‐19 group when compared with noncomplicated one. This result was also approved by previous studies done by Yao et al.^32^ and Gao et al.^33^

In this study, the univariate analysis detected that d‐dimer level between 1 and 2 µg/ml was a significant risk factor for the disease severity (OR = 11.8 [95% CI = 3.26–42.8]). Zhou et al.^34^ reported that mortality risk increased with a d‐dimer level of more than 1 μg/ml (OR 10.17 [95% CI = 1.10–94.38]). The cytokine storm leads to vascular endothelial damage, stimulating the coagulation system with inhibition of the fibrinolytic system.^28^

In this study, hyperferritinemia was observed in patients with the severe complicated disease on admission and significantly increased among them as compared to the mild noncomplicated patients. Also serum ferritin level was one of the significant risk factors for COVID‐19 infection severity in both the univariate and multivariate analysis. Moreover, in our univariate analysis, in‐hospital death odds were elevated in cases with higher serum ferritin levels. Similarly, Ji et al.^35^ observed that patients with nonsevere disease have serum concentrations of ferritin generally within the normal range. Also, they found that patients with the severe disease on admission had a higher level of ferritin than 400 μg/L. In addition, another study reported 1.5–5.3 times higher levels of ferritin levels on ad‐mission among severely‐ill COVID‐19 patients than in less‐severe cases.^36^ In parallel, serum ferritin was described as an independent predictor for COVID‐19 severity.^37^

Some limitations of this study are notably apparent. First, causality interpretation is difficult due to the utilization of a cross‐sectional study design; however, interpretation of the regression results might be of relevance. Second, internal validity could be decreased due to the convenient sampling method which is subjected to selection bias as compared to the random sampling technique.

## 5 CONCLUSION

This study revealed that elevated levels of d‐Dimer, serum ferritin, CRP, HR, lower SpO_2_%, and lymphopenia aid in the risk of COVID‐19 patients' assessment and adequate management.

## AUTHOR CONTRIBUTIONS

*Idea conceiving, data collection, and writing the original draft*: Walaa A. Fadda. *Conceptualization, methodology writing, writing original draft, editing the manuscript, and conducting data analysis and management*: Manal A. Al‐Batanony. *Conceptualization, writing original draft, methodology supervision, and revising the drafts*: Reham E. E. Aboukhalil. *Conceptualization and writing – review*: Heba F. Khader. *Supervision, conceptualization, and revising the whole manuscript*: Osamah Al Rugaie. All authors contributed to the article and approved the submitted version.

## CONFLICT OF INTEREST

The authors declare no conflict of interest.

## ETHICS STATEMENT

This study was conducted in accordance with the Declaration of Helsinki and approved by the Committee of Research Ethics, Deanship of Scientific Research, Qassim University (approval code: 20‐06‐16 and date of approval: March 24, 2021). Informed consent was obtained from all subjects involved in the study. Written informed consent has been obtained from the patients to publish this paper.

## Data Availability

Data are available on reasonable request.
